# Dual-Mechanism Gastroretentive Tablets with Encapsulated Gentian Root Extract

**DOI:** 10.3390/pharmaceutics17010071

**Published:** 2025-01-07

**Authors:** Jelena Mudrić, Ljiljana Đekić, Nemanja Krgović, Đorđe Medarević, Katarina Šavikin, Milica Radan, Nada Ćujić Nikolić, Tijana Ilić, Bojana Vidović, Jelena Đuriš

**Affiliations:** 1 Institute for Medicinal Plants Research “Dr. Josif Pančić”, 11000 Belgrade, Serbia; nkrgovic@mocbilja.rs (N.K.); ksavikin@mocbilja.rs (K.Š.); mradan@mocbilja.rs (M.R.); ncujic@mocbilja.rs (N.Ć.N.); 2 Faculty of Pharmacy, University of Belgrade, 11221 Belgrade, Serbia; ljiljana.djekic@pharmacy.bg.ac.rs (L.Đ.); djordje.medarevic@pharmacy.bg.ac.rs (Đ.M.); tilic@pharmacy.bg.ac.rs (T.I.); bojana.vidovic@pharmacy.bg.ac.rs (B.V.); jelena.djuris@pharmacy.bg.ac.rs (J.Đ.)

**Keywords:** double emulsion, solid lipid microparticles (SLMs), quality by design, trehalose, in vitro digestion, gentiopicroside, *Gentiana lutea*, biphasic release, floating, mucoadhesion

## Abstract

**Background/Objectives:** This study aimed to develop gastroretentive tablets based on mucoadhesive–floating systems with encapsulated gentian (*Gentiana lutea*, Gentianaceae) root extract to overcome the low bioavailability and short elimination half-life of gentiopicroside, a dominant bioactive compound with systemic effect. The formulation also aimed to promote the local action of the extract in the stomach. **Methods:** Tablets were obtained by direct compression of sodium bicarbonate (7.5%) and solid lipid microparticles (92.5%), which were obtained with lyophilizing double emulsions. A quality by design (QbD) was employed to evaluate the impact of formulation factors and processing parameters on emulsion viscosity, powder characteristics (moisture content, encapsulation efficiency, flowability), and tablet characteristics (floating lag time, gentiopicroside release, and assessment of dispersibility during in vitro dissolution). **Results:** The trehalose content and high-shear-homogenization (HSH) time of primary emulsion were critical factors. Trehalose content positively influenced emulsion viscosity, moisture content, floating lag time, encapsulation efficiency, and the release rate of gentiopicroside. HSH time positively affected powder stability and negatively gentiopicroside release. The selected powder had a high gentiopicroside encapsulation efficiency (95.13%), optimal stability, and good flowability. The developed tablets exhibited adequate floating lag time (275 s), mucoadhesive properties, and gentiopicroside biphasic release (29.04% in 45 min; 67.95% in 6 h). Furthermore, the optimal tablet formulation remained stable for 18 months and was primarily digested by duodenal enzymes. **Conclusions:** Dual-mechanism gastroretentive tablets with encapsulated gentian root extract were successfully developed. The in vitro digestion study demonstrated that the optimal formulation effectively resisted gastric enzymes, ensuring the release of its contents in the small intestine, even in the case of premature gastric evacuation.

## 1. Introduction

The oral administration route is the preferred approach for administering bioactive compounds due to its well-known advantages, such as noninvasive nature, high patient compliance, and cost-effectiveness. However, bioactive compounds are often not effective in conventional dosage forms due to their low stability and/or bioavailability [1]. Therefore, various delivery systems have been developed according to different patients’ needs and specific biopharmaceutical issues. Gastroretentive delivery systems were designed to be retained in the upper part of the gastrointestinal tract for a prolonged period of time, during which they release the bioactive compound in a controlled manner [2,3]. Several strategies have been developed to prolong gastric residence time. Floating systems are gastroretentive systems with a density lower than that of gastric contents, allowing them to remain buoyant in gastric fluids and release the drug over an extended period. According to previous studies, floating systems seem to be those with the best perspectives [2]. However, their performance can be influenced by floating lag time, variations in gastric fluid volume, food intake, and the presence of other gastric contents. Swelling or expandable systems rapidly increase in size to prevent passage through the pylorus, but they often require large amounts of swellable polymers, making them less practical for high-dose active ingredients and more challenging to manufacture due to high costs. Mucoadhesive systems adhere to the gastric mucosa, resisting peristaltic movements, but their effectiveness can vary depending on local pH and mucus turnover. High-density systems, which incorporate excipients like barium sulfate, sink to the bottom of the stomach, though they are not ideal for delivering large doses of sensitive materials. Magnetic systems use an embedded magnet and an external magnetic field to retain the dosage form in the stomach, but their practical applicability is limited [2,4].

Mucoadhesive–floating drug delivery systems are designed to improve the gastric residence of active compounds through a combination of mucoadhesion and floating mechanisms. The rationale behind combining floating and mucoadhesion strategies is that each approach independently prolongs gastric residence time through different mechanisms. By integrating both strategies into a single dosage form, the system can remain close to the absorption site more reliably, even when floating alone might not suffice. Studies have shown that mucoadhesive–floating systems improve gastric retention, leading to enhanced bioavailability and a prolonged half-life for various drugs [4,5,6,7,8,9]. This approach is particularly relevant for active compounds requiring extended contact time in the stomach or upper intestine to achieve adequate therapeutic outcomes.

Plant extracts are often used in relatively high doses and are typically in a liquid state before further processing. Many existing floating or mucoadhesive platforms rely on organic solvents or are best suited for smaller-dose actives. Our technique addresses this gap by enabling the incorporation of a bulk liquid extract into a solid dosage form without the use of organic solvents, thus facilitating the development of a mucoadhesive–floating system suitable for herbal products. Namely, lipid-based floating systems with Gelucire^®^ 43/01 are promising due to their low-density and triglyceride-based nature that promotes contact with the intestinal membranes and increases the solubilization, while at the same time, gastric emptying is reduced, and thus, the absorption of bioactive compounds is improved. Furthermore, the low melting temperature of this lipid is considered as favorable during the processing of delivery systems for unstable bioactive compounds [10,11,12].

Gentian (*Gentiana lutea* L., Gentianaceae) root extract is used as a traditional herbal medicinal product for temporary loss of appetite and for the treatment of dyspeptic/mild gastrointestinal complaints. However, gentiopicroside, a dominant bioactive compound with significant pharmacological potential, is characterized by low bioavailability and a short elimination half-life, which limit its therapeutic efficacy. These pharmacokinetic challenges necessitate the development of modified-release formulations to optimize its absorption, prolong systemic circulation, and enhance therapeutic outcomes [13,14]. In addition, the extract has systemic activity and local effects in the stomach, so prolonged release in the stomach could improve the therapeutic effect and reduce the dosing frequency. Therefore, gentian root extract is used as a model active compound during the formulation of lipid-based gastroretentive delivery systems. Taking into account the hydrophilic nature of gentiopicroside and many other bioactive compounds from gentian ethanolic extract [15], the incorporation of gentian extract in a lipid-based gastroretentive system could be challenging. In our previous study, encouraging results (yield > 90% and encapsulation efficacy > 90%) were obtained by employing the double emulsion–melt dispersion technique and subsequent lyophilization [11].

However, there are many material attributes and process parameters that could influence the quality of lipid-based gastroretentive systems loaded with gentian extract obtained through the lyophilization of double emulsions. Therefore, the quality by design (QbD) approach is promising approach for developing lipid-based gastroretentive tablets with a high encapsulation efficiency and tailored release of gentiopicroside. This process should be conducted taking into account the key assertion of QbD that quality is not controlled by simply testing the product, but rather by building quality into the formulation and manufacturing process [16].

The aim of this study was to develop lipid-based gastroretentive tablets with encapsulated gentian root extract and to systematically evaluate the impact of formulation factors and processing parameters on the quality attributes by conducting a comprehensive characterization and risk assessment. Furthermore, since lipid-based formulations are substrates for digestive lipases, in vitro digestion was conducted in order to assess the influence of digestion on the release of gentiopicroside from optimal gastroretntive formulation.

## 2. Materials and Methods

### 2.1. Materials

Gentian (*Gentiana lutea*, Gentianaceae) roots (batch number: 19910718910) were obtained from the production sector of the Institute for Medicinal Plants Research “Dr. Josif Pančić” (Belgrade, Serbia). Polyglycerol polyricinoleate—PGPR (Palsgaard, Juelsminde, Denmark) was an W/O emulsifier, while soybean lecithin Lipoid S 75 (Lipoid GmbH, Ludwigshafen, Germany) was used as a O/W emulsifier in the preparation of W/O/W emulsions. Gelucire^®^ 43/01 (Gattefossé, Saint-Priest, France) was utilized as a lipid component and release-retarding agent. Trehalose dihydrate (Tokyo Chemical Industry, Tokyo, Japan) was incorporated as a cryoprotectant. Biopolymer sodium alginate (Thermo Fisher Scientific, Waltham, MA, USA) was incorporated as a thickening agent in order to improve the thermodynamic stability of the double emulsion, as well as the mucoadhesive properties of the tablets. Sodium chloride was included in the formulation in order to improve their thermodynamic stability (Sigma-Aldrich, Münich, Germany). Sylysia^®^ 350, highly porous micronized silica (Fuji Silysia Chemical, Kasugai Aichi, Japan), was incorporated in the formulation to improve the tablets’ mechanical properties. For the mucoadhesion evaluation, the mucin from a porcine stomach, Type II (Sigma-Aldrich, Shanghai, China), was used. Methylcellulose (Metolose^®^ SM 4000, Shin-Etsu Chemical, Tokyo, Japan) was used to mimic the rheological properties of human gastric fluid during the procedure.

The digestion procedure was conducted using rabbit gastric extract (Lipolytech, Marseille, France), porcine pancreatin (Sigma-Aldrich, St. Louis, MO, USA), bovine bile (Sigma-Aldrich, St. Louis, MO, USA), and all other reagents according to the INFOGEST protocol [17]. Reagents used for the assessment of antioxidant activity, including Trolox (97%), 2,4,6-tris (2-pyridyl)-s-triazine, ferric chloride hexahydrate, sodium acetate, 2,2ʹ-diphenyl-1-picrylhydrazyl, Tween^®^ 20, linoleic acid, and β-carotene, were purchased from Sigma-Aldrich, St. Louis, MO, USA. All other reagents were of analytical grade, including orthophosphoric acid (Sigma-Aldrich, Münich, Germany), acetonitrile (Merck, Darmstadt, Germany), ethanol, methanol, hydrochloric acid, and glacial acetic (Thermo Fisher Scientific, Waltham, MA, USA). Ultra-pure water was obtained using a Milli-Q purification system (Millipore, Guyancourt, France). The gentiopicroside standard was provided by ChromaDex (Los Angeles, CA, USA).

### 2.2. Liquid Gentian Extract Preparation

Gentian extract was obtained under the same conditions as previously described [11]. Ethanol (50%, *v*/*v*) was used as a solvent, and the solid-to-solvent ratio was 1:2 (g/mL). Ethanol was evaporated from the filtrated extract with a rotary vacuum evaporator (HB4-basic, IKA, Germany).

### 2.3. Risk Assessment Analysis

An Ishikawa fish-bone diagram was created to identify various material attributes (MAs) and process parameters (PPs) that can influence the quality, safety, and efficacy of gastroretentive tablets prepared through the direct compression of powders obtained through the lyophilization of the double (W/O/W) emulsion. For the selection of critical risk factors with the highest impact on critical quality attributes (CQAs), a risk estimation matrix (REM) was created by assigning low, medium, and high ranks to expected risks [18,19].

### 2.4. Preparation of W/O/W Emulsions

Double emulsions (W/O/W) were fabricated according to the multiple (double) emulsion–melt dispersion technique and characterized using methods reported by Mudrić et al. [11]. Briefly, the heated inner water phase was added to the melted oil phase at 48–53 °C (Table 1). Primary (W/O) emulsions in the case of formulations H and J were homogenized at 15,000 rpm for 3 min, while emulsions G and I were homogenized at 15,000 rpm for 6 min using a high-shear homogenizer (Ultra-Turrax, IKA^®^, Staufen, Germany). In the next step, the primary emulsion (W/O) was dispersed in the outer water phase. The trehalose content in prepared emulsions G and H was 3.94%, while in emulsions I and J, the content was 7.88%. Sylysia^®^ 350 was added after the homogenous dispersion formation, and emulsions were homogenized at 3000 rpm for 4.5 min with a high-shear homogenizer. After that, a laboratory mixer continually stirred the sample until the temperature of the prepared emulsion was approximately 25 °C.

### 2.5. Characterization of W/O/W Emulsions

Emulsions were prepared and stored at room temperature for 24 h prior to the rheological characterization. Microscopic analysis was performed on an optical microscope (Olympus BX41 with camera Olympus SC30, Olympus, Tokyo, Japan) after diluting samples with purified water. The conductivity was measured using a conductivity meter (Radiometer, Copenhagen, Denmark). The samples’ pH was measured using a pH meter HI 9321 (Hanna Instruments, Woonsocket, RI, USA). The stability of emulsions was analyzed by conducting a centrifugation test (15,000 rpm for 15 min at 22 ± 2 °C).

Rheological measurements were carried out on a rotational and oscillatory rheometer Rheolab MC 120 (PaarPhysica, Austria) coupled with a cone and plate measuring system MK 22 with a cone diameter of 50 mm, cone gap of 50 µm, and cone angle of 1°. The measurements were performed at 20 ± 0.1 °C. The controlled shear rate (CSR) procedure was applied for a flow curve construction by increasing the shear rate from 0 s^−1^ to 200 s^−1^ and decreasing it back from 200 s^−1^ to 0 s^−1^. Each interval data point (upward and downward) comprises 30 measurement points, and the measurement point duration was 10 s.

### 2.6. Powder Preparation

Powders (G, H, I, and J) were obtained through the lyophilization of double emulsions (G, H, I, and J) at the PVP Centre for Lyophilization (Valjevo, Serbia) as previously described [11]. The theoretical compositions of the powders after lyophilization are presented in Table 1. Gentian extract powder was obtained through the lyophilization of liquid gentian extract, under the same conditions as emulsions.

### 2.7. Scanning Electron Microscopy (SEM)

The morphology of powders obtained through the lyophilization of double emulsions and gentian extract powder were analyzed using a JEOL JSM-6390LV scanning electron microscope (JEOL USA, Inc., Peabody, MA, USA). Before the SEM analysis, powders were coated with gold for 100 s under 30 mA ion current.

### 2.8. Differential Scanning Calorimetry

Differential scanning calorimetry (DSC) of powder samples and gentian extract was performed using the DSC1 instrument (Mettler Toledo, Giessen, Germany). Accurately weighted powders were placed in aluminum pans and subjected to heating at 10 °C/min in the range from −60 to 200 °C under constant nitrogen gas flow (50 mL/min). An empty standard aluminum pan was a reference.

### 2.9. Fourier Transform Infrared (FTIR) Spectroscopy

The structural characterization of the prepared SLM powders (G, H, I, J), along with the dry gentian extract, was performed utilizing an Agilent Technologies Cary 630 FTIR spectrometer equipped with the Diamond-ATR sampling module. The spectral analysis was executed in the wavenumber range from 4000 cm^−1^ to 500 cm^−1^ with a resolution of 8 cm^−1^.

### 2.10. Yield

The yield was expressed as a percentage of the obtained microparticle mass with respect to total solids used for the preparation of emulsions.

### 2.11. Determination of the Encapsulation Efficiency

The gentiopicroside encapsulation efficiency (EE) was determined initially after preparation and after 18 months of storage at room temperature, as previously reported by Mudrić et al. [11]. Concisely, the samples were sonicated for 30 min at 70 ± 5 °C in the water bath. The content of gentiopicroside in the samples was determined by high-performance liquid chromatography (Section 2.12.), and the EE was calculated initially after preparation according to Equation (1) and after 18 months of storage according to Equation (2).

EE = (Actual quantity of gentiopicroside entrapped in powder)/(Theoretical quantity of gentiopicroside in powder) × 100 (1)

EEafter 18 months = (Actual quantity of gentiopicroside entrapped in powder after 18 months)/(Actual quantity of gentiopicroside entrapped in powder initially) × 100(2)

### 2.12. High-Performance Liquid Chromatography

The gentiopicroside content in the samples was analyzed on Agilent 1200 RR HPLC instrument (Agilent, Waldbronn, Germany), on a reverse-phase analytical column Zorbax SB-C18 (Agilent, Waldbronn, Germany), as previously reported [11].

### 2.13. Moisture Content Analyze

The moisture content of the samples was measured gravimetrically using the Halogen Moisture Analyzer HB43-S (Mettler Toledo, Switzerland).

### 2.14. Floating Behavior

The floating lag time and floating duration of powders and tablets were determined in a water bath at 50 rpm and 37 ± 0.5 °C. Samples were placed in a glass beaker, containing 200 mL of 0.1 M HCl and 0.2% methylcellulose to mimic the rheological properties of human gastric fluid [20]. The floating lag time was recorded as the time between sample introduction and its buoyancy, while floating duration was measured as the time during which the sample remains buoyant.

### 2.15. Evaluation of Powder Flowability

The Hausner ratio and the Carr index were calculated according to Equations (3) and (4), respectively, to assess powders’ flowability. Firstly, the unsettled bulk volume (V_0_) of a powder sample with a known weight was determined. After that, the tapped volume (V_f_) was measured after the tapping sample with a tapped density tester (Stampfvolumeter, STAV 2003, Jel, Ludwigshafen, Germany) according to European Pharmacopoeia 11.0 (Ph. Eur. 11.0) [21].

HR = V_0_/V_f_ (3)

CI = 100 × ((V_0_ − V_f_)/V_0_) (4)

### 2.16. Antioxidant Activity

A comparison of the antioxidant activity of the prepared powders (G, H, I, and J) after 12 months of storage and fresh liquid gentian extract was carried out in order to estimate the influence of processing and storage conditions on the antioxidant activity. Three in vitro assays were conducted: ferric-reducing antioxidant power (FRAP), 2,2′-diphenyl-1-picrylhydrazyl radical scavenging activity (DPPH), and β-carotene bleaching inhibition. The antioxidant potency composite index (ACI) was calculated by assigning all the antioxidant tests an equal weight. For each test, ab index value of 100 was attributed to the liquid gentian extract. The average of all three tests was reported as the ACI. A microplate reader (BIOTEK, Santa Clara, CA, USA) was used for the measurement of the investigated samples’ absorbance.

### 2.17. FRAP Assay

This assay was conducted according to the method described by Benzie and Strain [22]. The samples’ absorbances were measured at 630 nm. The results were expressed as µmol of Trolox equivalents (TE) per g of sample (µM TE/g) by applying a calibration curve constructed using a standard (Trolox, 100–800 µM).

### 2.18. DPPH Assay

The radical scavenging activities of the investigated powders and liquid gentian extract against DPPH radicals were estimated following the method described by Brand-Williams et al. [23]. The absorbances of samples were measured at 490 nm. The results were expressed as µM of Trolox equivalents (TE) per g of sample (µM TE/g) by applying a calibration curve constructed using a standard (Trolox, 200–700 µM).

### 2.19. Beta-Carotene Bleaching Assay

The antioxidant activity of the investigated samples against lipid peroxyl radical was determined according to the method reported by Reis et al. [24]. The samples’ absorbance was measured at 450 nm immediately after the addition of the β-carotene/linoleic acid emulsion, as well as after the incubation period. The results were reported as a percentage of β-carotene bleaching inhibition.

### 2.20. Tablet Preparation

Four tablet formulations, G, H, I, and J, were prepared by mixing 92.5% of the prepared powders (G, H, I, and J) and sodium bicarbonate (7.5%). The theoretical composition of powders G, H, I, and J is shown in Table 1. Tablets were obtained through the direct compression of tablet formulations G, H, I, and J (approximately 100 mg) on a benchtop single-punch tablet press Gamlen D series (Gamlen Tableting Limited, London, UK). The same flat punch (6 mm), load (30 kg), compaction speed (60 mm/min), and dwell time (0.08 s) were applied in the case of all formulations.

### 2.21. Friability Testing

The friability of the tablets was determined according to European Pharmacopoeia 11.0 [21]. Briefly, accurately weighted and dedusted tablets were rotated (25 ± 1 rpm) in the drum for four min. After that, the dust was removed, and the tablets were weighed. The friability was expressed as a percentage of weight loss.

### 2.22. Mucoadhesion Evaluation

The mucoadhesion of tablets was determined as a force required to separate the investigated tablet (approximately 100 mg) from the raw gastric porcine mucin (250 mg) according to the procedure reported by Mudrić et al. [11]. The force of adhesion was measured on a Texture Analyzer Shimadzu EZ-LX (Shimadzu Corporation, Kyoto, Japan), with a 5 kg load cell and 10 mm aluminum cylindrical probe. A force of 0.5 N was applied on the tablet for 60 s to ensure intimate contact between the tablet and the mucin disk, where the pre-test speed was 1 mm/s, the test speed was 0.5 mm/s, and the post-test speed was 0.5 mm/s. The obtained force–time curves were utilized to determine the force of adhesion as an indicator of tablet mucoadhesion.

### 2.23. In Vitro Gentiopicroside Release Testing

Tablets were tested using a USP IV apparatus (Flow-through cell, CE7 smart, Sotax, Aesch, Switzerland) to evaluate the release kinetics of gentiopicroside, as previously described [11]. The temperature of the dissolution medium (0.1 M HCl, pH 1.2, 100 mL) was 37 ± 0.5 °C during 6 h, and the flow rate was 8 mL/min. Small volumes (approximately 2.5 mL) of medium were sampled at fixed time intervals (15, 45, 90, 150, 240, 360 min) and immediately replaced with an equal amount of fresh prewarmed medium. Pooled samples were prepared according to the procedure for botanical dosage forms described at the United States Pharmacopeial Convention [25]. The concentration of dissolved gentiopicroside in those samples was determined by high-performance liquid chromatography (Section 2.12.). In addition, the comparison of gentiopicroside release profiles from all tablet formulations and dry gentian extract powder was carried out with a model-independent index described by Moore and Flanner [26], known as the similarity factor (*f*_2_), according to Equation (5). A value of *f*_2_ lower than 65 implies that the profiles are significantly different, while a value of *f*_2_ in the range from 65 to 100 indicates a similarity between the profiles of over 95% [27].

(5) $$f_{2}=50\times log\left\{{\left[1+\frac{1}{n}\sum_{n=1}^{t}{\left(R_{t}-T_{t}\right)}^{2}\right]}^{-0.5}\times 100\right\}$$

In the Equation (5) *n* is the number of dissolution sampling times, and *R_t_* and *T_t_* are the gentiopicroside release percentage at each time point for the reference and test sample, respectively.

### 2.24. Assessment of Dispersibility During In Vitro Release Testing

During the in vitro release testing of investigated tablet formulations (G–J) the average size of the dispersed nanostructures (0.1 M HCl) was measured after 15 min and after 6 h, using photon correlation spectroscopy. The measurements were performed at 20 ± 0.1 °C using the Zetasizer NanoZS90 instrument (Malvern Instruments, Malvern, UK) with the integrated He-Ne laser at 633 nm and scattered light detector at 90 °C.

### 2.25. In Vitro Digestion

The in vitro digestion of optimal sample was conducted according to the INFOGEST protocol [17]. Firstly, enzyme activity assays were carried out. The experiment was performed in a shaking water bath at 37 ± 0.5 °C with a shaking frequency of 40 rpm/min. The simulated digestion fluids for the oral (SSF), gastric (SGF), and small intestinal phase (SIF) were prepared with NaCl instead of NaHCO_3_ to avoid the formation of bubbles, as described in the INFOGEST protocol.

The adequate sample mass was calculated by considering a single dose of liquid gentian extract (1 g) and estimated intestinal fluid volume (200 mL) [13,28]. Accurately weighted samples with a mass of approximately 100 mg were added to SSF (1.5 mL) that was prewarmed to 37 °C. The enzyme (α-amylase) was not included in the oral phase as the investigated samples are usually swallowed immediately. After the incubation (30 s) at 37 °C, oral bolus was added to prewarmed SGF. Also, rabbit gastric extract (RGE) was added to achieve lipase activity of 60 U/mL in the final gastric mixture. Finally, a gastric chime was added to the prewarmed SIF. Consequently, the bile solution was mixed with the prepared sample in order to reach a final concentration of 10 mM. Pancreatin was added to this mixture to achieve a lipase activity of 2000 U/mL since the lipid-based formulation was investigated. To stop lipase activity in the gastric phase, pH was raised to 8, while in the intestinal phase, orlistat (tetrahydrolipstatin) with a final concentration of 5 mM was used. In order to examine the influence of enzymes, control samples were investigated under same conditions, but without enzymes and bile salts.

### 2.26. Statistics

The influence of critical factors on the CQA was estimated using two-way ANOVA. Statistical analysis was performed using the SPSS (trial version). Statistical significance was designated as *p* < 0.05. All experiments were carried out in three replications, and the obtained results are presented as the mean value ± standard deviation (S.D.).

## 3. Results and Discussion

### 3.1. Identification of QTPP and CQAs

Quality target product profile (QTPP) was developed, taking into account the safety and the efficacy of the lipid-based gastroretentive tablets with gentian extract (Table 2), according to the ICH Guideline [16]. In the next step, critical quality attributes were defined as in Table 3.

### 3.2. Risk Assessment

**Figure 1:**
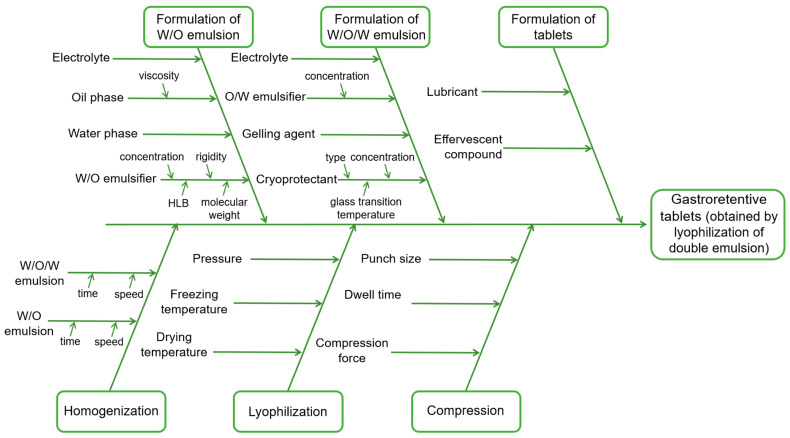
Ishikawa diagram illustrating the parameters potentially influencing the quality of lipid-based gastroretentive tablets.

The risk assessment analysis was based on previous results, knowledge, experience, and relevant information about lipid-based gastrorentive solid dosage forms [11,19,36,37,38]. An Ishikawa fish-bone diagram is presented in Figure 1 to elucidate the cause–effect relationship among the various material attributes (MAs) and process parameters (PPs), and the expected consequence of these on the CQAs.

Moreover, the REM carried out for the qualitative analysis of the risk associated with material attributes and process parameters is illustrated in Table 4. The REM suggested that factors with the highest impact on the examined CQAs are trehalose content and high-shear-homogenization (HSH) time. Furthermore, it was estimated that HSH time during O/W emulsion preparation has a higher impact on the final product quality than in the case of W/O/W emulsion preparation, since W/O/W emulsion is processed under lower speeds. Therefore, the impacts of trehalose content and W/O emulsion HSH time were further investigated.

Namely, since the stresses during lyophilization create irregular ice crystals that could result in a porous microstructure that might influence entrapment efficiency, selecting the suitable type and concentration of cryoprotectant is essential. Cryoprotectants used during double emulsion lyophilization are mainly monosaccharides and disaccharides, whereas trehalose has numerous advantages due to its high Tg (glass transition temperature) and its potential to create intermolecular hydrogen bonds [39,40]. It was reported previously that trehalose content influenced the viscosity of emulsions, size, and structure of particles, as well as the release rate of proteins and various active compounds such as bendroflumethiazide and levonorgestrel. Moreover, the stability of bioactive compounds (β-carotene) was influenced by trehalose content in the case of physical mixtures, as well as various delivery systems [41,42,43,44,45]. Furthermore, the influence of trehalose content on gastroretentive systems was not investigated previously. Thus, delivery systems obtained through the lyophilization of double emulsion with 7.88% and 3.94% trehalose were formulated and compared. Also, it is demonstrated that the HSH process could influence emulsion rheological properties, the morphology particle size of delivery systems, and, consequently, the encapsulation efficiency, as well as stability [46,47,48]. Therefore, HSH time and speed are important factors, and in the preliminary study, it was estimated that the optimal speed during W/O processing was 15,000 rpm. The HSH process produces cavitation, collision, and turbulence forces, which cause the breakdown of the droplets. Prolonged homogenization times may induce instability in colloidal systems due to the excessive input of energy that may provoke coalescence due to an increase in the surface area, thus favoring the formation of systems with a high polydispersity index. On the other hand, insufficient homogenization duration may fail to generate stable double emulsions. The optimal HSH time ensures the application of appropriate energy, promoting the formation of a stable colloidal system with uniform size distribution, and consistent morphology after lyophilization, as well as enhanced stability after production. Consequently, the effect of homogenization time on CQA should be investigated. Therefore, W/O emulsions homogenized for 3 and 6 min were investigated.

### 3.3. Emulsions

Upon preparation and after seven days of refrigeration, the formulated emulsions (G, H, I, and J) exhibited a consistent yellow color and homogeneous appearance. Moreover, after the centrifugation test, all tested samples were homogenous without a change in consistency. The conductivity of analyzed samples was in the range of 4.25 to 5.54 µS/cm (Table 5), indicating that the water phase was the external phase of emulsions [49]. The investigated emulsion pH was from 4.39 to 4.46.

The generation of double (W/O/W) emulsions was confirmed by microscopic analysis in the case of all samples (Figure S1). It was observed that double emulsions with complex inner structures were formulated. This type of double emulsion, known as the microsphere type, is generally desirable due to the high encapsulation efficiency and good stability of developed emulsions [50].

**Figure 2:**
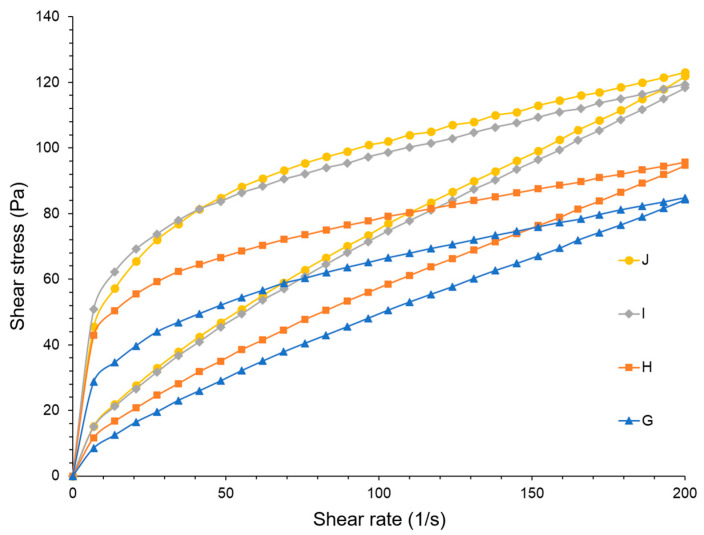
Hysteresis loops of the double emulsions.

Figure 2 represents the upward and downward flow curves of double emulsions G, H, I, and J. All investigated double emulsions showed non-Newtonian shear thinning (i.e., pseudo-plastic) flow behavior with a hysteresis loop between the upward and downward flow curves. The extent of thixotropy of the samples is expressed as a hysteresis area (HA). The measured values of the maximal apparent viscosity (η_max_), minimal apparent viscosity (η_min_), and calculated values of HA are shown in Table 5.

**Figure 3:**
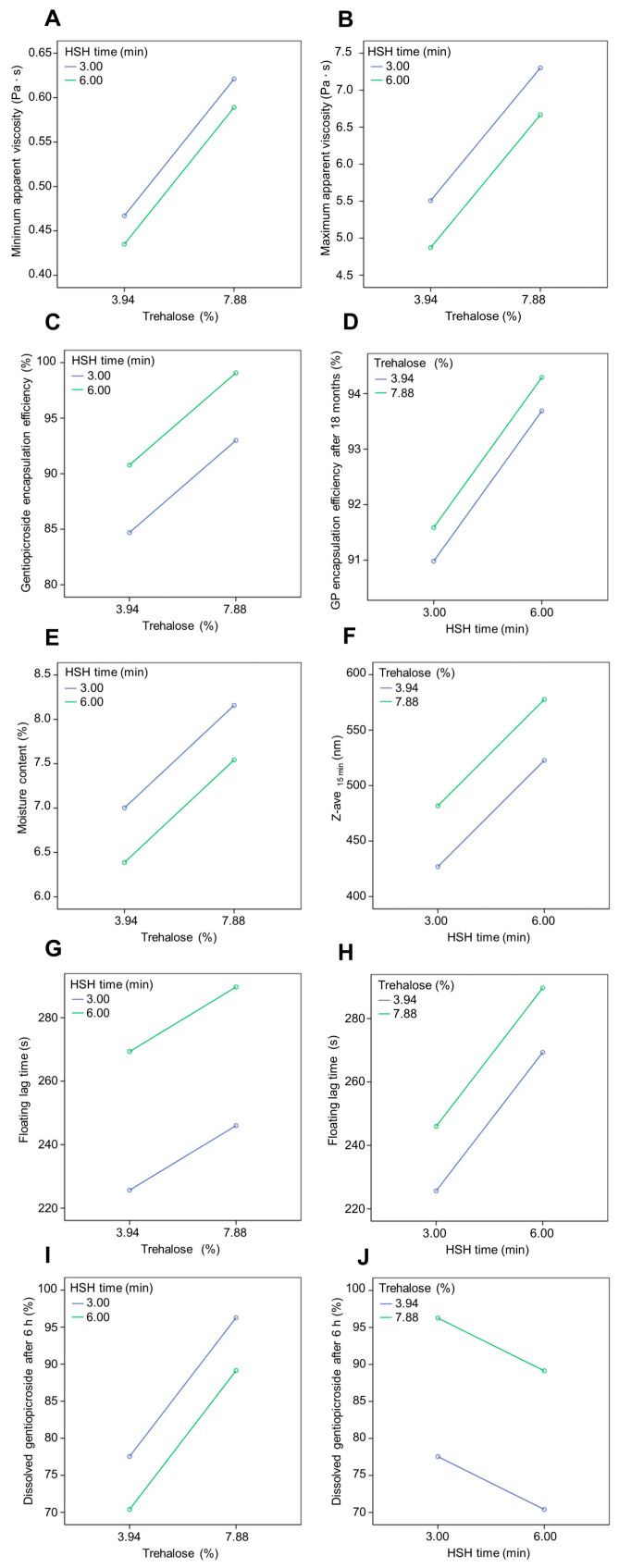
Influence of high-shear-homogenization (HSH) time and trehalose concentration on critical quality attributes i.e., (**A**) minimal apparent viscosity; (**B**) maximal apparent viscosity; (**C**) gentiopicroside encapsulation efficiency; (**D**) gentiopicroside encapsulation efficiency after 18 months; (**E**) moisture content; (**F**) Z-average; (**G**,**H**) floating lag time; (**I**,**J**) dissolved gentiopicroside after 6 h.

For all double emulsions, it was observed that the apparent viscosity decreased with an increasing shear rate from the maximum value to the minimum value (Figure 2). After a gradual decrease in shear rate to 0 s^−1^, the apparent viscosity values were equal to the initial values. This reflected a time-dependent process of the temporary disturbance of the internal structure of the dispersion system and its recovery after the termination of the action of the shear stress, whereby the observed thixotropy indicated that the recovery process was slower than the structure disturbance [51]. This type of rheological behavior is typical for double emulsions that contain a thixotropic water-soluble polymer in their outer water phase [52], whereby the thixotropic character of the investigated double emulsions was attributed to sodium alginate, which was added to both the external and internal water phase (Table 1). The comparison of the maximum and minimum values of the apparent viscosity of the investigated double emulsions showed the increasing order as follows: G ˂ H ˂ J ˂ I. Also, in the same order as the double emulsions, the hysteresis area, as a measure of thixotropy of the system, ranges from 2963.45 Pa/s (G) to 4932.91 Pa/s (I). It follows that formulation G showed the lowest extent of the disruption of the polymer network within the external water phase and the relatively fastest recovery under the conditions of the performed rheological measurement, while emulsion I showed the highest extent of the polymer network disruption and the slowest recovery, although the concentration of sodium alginate in all emulsions was the same (Table 1). In addition, it was observed that the values of the minimum and maximum apparent viscosity and hysteresis area of formulations G and H, with lower trehalose content (3.94%), were lower compared to those of formulations I and J, comprising higher trehalose concentration (7.88%). Statistical analysis showed that trehalose content was an important parameter (Figure 3A,B) that positively affected the maximum apparent viscosity (*p* = 0.004) and minimum apparent viscosity (*p* = 0.000), while the HSH time did not significantly affect the investigated parameters (*p* = 0.215; *p* = 0.054, respectively). This result follows previous findings since it has been reported that viscosity increases proportionally to trehalose concentration in aqueous solution [53].

Domian et al. [54] showed that O/W emulsions with trehalose in the concentration range 6–26% were shear-thinning non-Newtonian fluids in the examined range of shear rates, which was in agreement with the results obtained for the investigated double emulsions G, H, I, and J. According to the available literature, in aqueous solutions, as well as in the external aqueous phase of O/W emulsions, trehalose forms hydrogen bonds with water above and below the glass transition temperature (Tg) [55,56]. Trehalose, as a disaccharide with the highest glass transition temperature (Tg), remains in the glassy state in a wider range of temperatures and the viscosity of the mentioned systems remains relatively high [57]. It can be assumed that in the outer water phase of the investigated double emulsions, trehalose formed the sugar–water matrix through hydrogen bonding, particularly at the higher trehalose content in formulations I and J, which led to the higher values of apparent viscosities and hysteresis area compared to formulations G and H.

### 3.4. Powders

**Figure 4:**
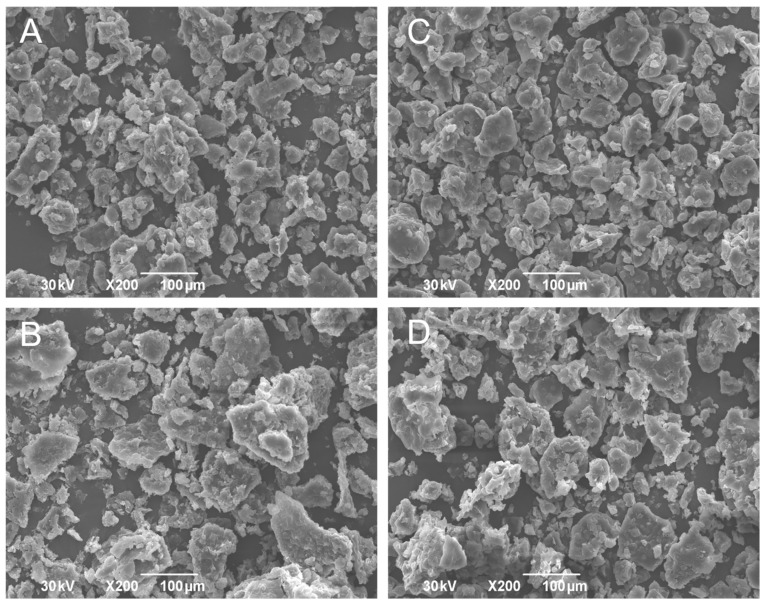
SEM images of solid lipid microparticles loaded with gentian extract: (**A**) powder G, (**B**) powder H, (**C**) powder I, and (**D**) powder J.

Powders prepared through the lyophilization of double emulsions were yellow and homogenous. SEM photomicrographs are presented in Figure 4. It was evident that amorphous powders with particle sizes under 1000 µm were obtained, suggesting that solid lipid microparticles (SLMs) were developed. The particles with flake-like shapes and porous structures were present in all investigated samples. According to the presented photomicrographs, a lipid matrix was formed after the drying of double emulsions G, H, I, and J, indicating that droplets formed in the double emulsions were structurally altered during the lyophilization process due to pressure and water evaporation, leading to the observed porous and flake-like morphology. It is reported that this shape is common for powders obtained by freeze-drying and subsequent grinding [11,58,59].

FT-IR analysis was performed considering the changes in the characteristic spectra of the gentian extract powder and SLM powders (I, J, G, H). The spectrum of the gentian extract showed several representative absorption bands corresponding to the predominant bioactive compounds. As presented in Figure S2A (Supplementary Materials), the -OH group stretching vibrations appear at 3272 cm^−1^, while peaks at 2929–2896 cm^−1^ are representative for C-H stretching vibrations. A double-bond stretching zone (C=O and C=C) includes peaks at wavelength region from 1729 cm^−1^ to 1606 cm^−1^, suggesting the presence of carbonyl functional groups and aromatic moieties among the abundant iridoids, predominantly gentiopicroside [60]. The intense absorption peaks centered at 1025 cm^−1^ and 987 cm^−1^ could be ascribed to the C-O stretching of the glucose skeleton [61]. FT-IR analysis of the SLM powders’ spectra revealed that they possess all representative vibrations from both gentian extract as well as Gelucire^®^ 43/01 (Figure S2A). However, changes in the intensity of the several characteristic adsorption bands of the gentian extract suggested the presence of interactions between the hydroxyl and carbonyl functional groups of the main bioactive components from gentian extract and the excipients. This finding is in accordance with previous results since it was reported that the C=O group could form hydrogen bonds with OH groups of Gelucire^®^ 43/01 [10].

A DSC thermogram (Figure S2B, Supplementary Materials) of freeze-dried gentian extract showed a broad endothermic melting peak in the range of temperatures (130–140 °C) which may correspond to the melting of gentiopicroside [62]. Further thermal events above 170 °C occurred due to thermal decomposition. DSC analysis of all freeze-dried formulations resulted in very similar thermograms. Sharp melting endotherm around 42 °C resulting from the melting of Gelucire^®^ 43/01 is clearly distinguishable on these thermograms. However, there are no melting endotherms that originate from either gentian extract or trehalose, indicating that components are dispersed homogenously in the amorphous form, and no crystallization occurred during freeze drying.

Furthermore, obtained powders were characterized in order to evaluate their encapsulation efficiency, yield, flowability, floating ability, residual moisture content, and stability, considering encapsulation efficiency and antioxidant stability after 18 and 12 months, respectively.

Powders G, H, I, and J were characterized by a very high yield, suggesting that an effective method and adequate materials were utilized (Table 6).

The gentiopicroside encapsulation efficiency was very high in the case of formulations G, I, and J, while formulation H was characterized by a slightly lower encapsulation efficiency (Table 6). Gentiopicroside, known as a rather unstable and highly hydrophilic compound [15,63], was successfully incorporated in SLM, indicating that a suitable process and formulation parameters were selected. This result could be explained by mild conditions during the encapsulation process, and the degradation of bioactive compounds was minimized. Furthermore, the selection of a suitable formulation enabled a high encapsulation efficiency. Gelucire^®^ 43/01 is a lipid with a heterogeneous nature (saturated polyglycolized glycerides consisting of mono-, di-, and triglycerides and mono- and di-fatty acid esters of polyethylene glycol) and consequently forms a lattice with structural imperfections and more free space that enables the encapsulation of the bioactive compounds [64]. Also, PGPR and lecithin were selected as suitable emulsifiers, as it was reported that this system could enable a high encapsulation efficiency and stability of the double emulsion, due to complementary rheological properties [11,38].

The obtained results (Figure 3C) confirm that the trehalose content (*p* = 0.000) significantly influenced the encapsulation efficiency of gentiopicroside in SLM powders, while the effect of HSH time was not significant (*p* = 0.068). Furthermore, this result is consistent with the reported statement that the more the cryoprotectant is used, the thicker the cryoprotectant layer will become, and finally, a higher encapsulation efficiency will be achieved in the case of SLM obtained by freeze-drying [65]. Although the effects of trehalose on bioactive compounds during lyophilization have been extensively studied, the mechanisms of cryoprotective effects of trehalose are extremely complex. One of the hypotheses suggests that the higher viscosity of the trehalose solution results in hindered fluctuations of molecules [57]. The results obtained in the present study are in line with this observation since the trehalose concentration positively influenced the viscosity of the double emulsion and, consequently, the encapsulation efficacy.

Previously, gentiopicroside and oleanolic acid were incorporated simultaneously into nanostructured lipid carriers, while the total encapsulation efficiency was 48.43% [53]. Also, gentiopicroside was loaded in PLGA nanospheres and m-PEG/PVP nanofibers intended for wound healing, and the encapsulation efficiency was 55.78–87.99% and 85.52%, respectively [66,67]. Another advantage of the incorporation of gentian extract instead of pure gentiopicroside is that gentiopicroside bioavailability is two times higher due to the presence of other bioactive compounds in the gentian extract [68].

Furthermore, it is shown that the gentiopicroside encapsulation efficiency was slightly lower after 18 months of storage at room temperature (Table 6), indicating that SLM powders with good stability were developed. Moreover, the results show that HSH time (*p* = 0.000) positively influenced the gentiopicroside encapsulation efficiency after 18 months, while the influence of trehalose content (*p* = 0.221) was not significant (Figure 3D). It can be assumed that the longer duration of the homogenization process enabled the incorporation of the gentian extract into the W/O/W emulsions and thus into the particle matrix, which contributed to better protection of the incorporated gentiopicroside. According to the literature, the slight decrease in gentiopicroside content is primarily due to oxidative and hydrolytic degradation, resulting from the synergistic effects of light and oxygen exposure [69,70]. In addition, the antioxidant activity of liquid gentian extract was compared with that of SLM powders after 12 months of storage. The results show that the antioxidant potency composite index was in the range of 86.45 to 95.20%, suggesting that the antioxidant activity of the extract was preserved during processing and storage.

The moisture content was in the range of 6.23 to 8.00%. Powders G and H obtained from emulsions with lower trehalose content, as well as with lower viscosity, were characterized with lower moisture content, and it was shown that trehalose content (*p* = 0.016) influenced the moisture content of powders (Table 6), while the influence of HSH time (*p* = 0.150) was not significant (Figure 3E). The same influence of viscosity was observed in the case of powders obtained by the spray drying of emulsions [71].

All four powders floated immediately in the simulated gastric fluid under continuous agitation and did not sink into the solution after 6 h, while dry gentian extract was dissolved immediately. This result could be related to the composition and porous structure of SLM. It is reported that the density of Gelucire^®^ 43/01 is 0.0856 g/cm^3^. The low density is an important property of floating powders, but at the same time, low density could negatively influence flowability and usability in the industrial setup [72]. The powders’ ability to flow freely is also crucial in order to ensure adequate filling during production and, consequently, the accurate dosing and uniform mass of the final product. According to the results presented in Table 6, all four SLM powders (G, H, I, and J) were characterized with good flowability according to European Pharmacopoeia 11.0 [21], while dry gentian extract exhibited poorer properties (Carr index: 21.64; Hausner ratio: 1.28). Furthermore, it was noticed that the influence of trehalose content (*p* = 0.398) and HSH time (*p* = 0.126) on the Carr index was not significant. The same trend was noticed in the case of the Hausner ratio, since the influence of trehalose content (*p* = 0.582) and HSH time (*p* = 0.121) was not significant.

### 3.5. Tablets

**Figure 5:**
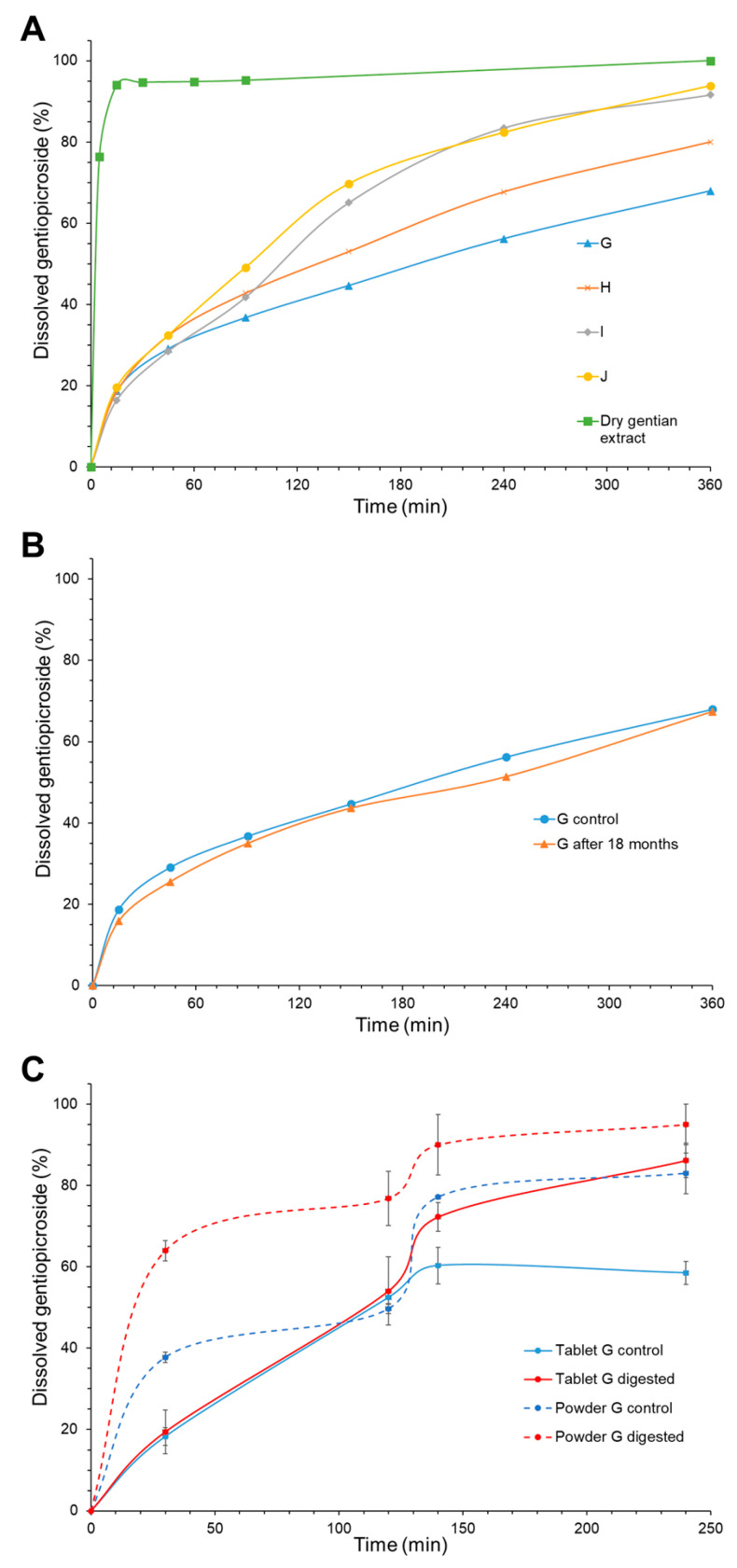
(**A**) Gentiopicroside dissolution profile from tablets (G, H, I, and J). (**B**) Comparison of gentiopicroside dissolution profile from tablet formulation G after preparation and after 18 months of storage. (**C**) In vitro digestion—gentiopicroside dissolution profile from tablet G.

Gastroretentive tablets were analyzed in order to evaluate their floating ability (floating lag time and total floating duration), friability, mucoadhesive properties, and gentiopicroside dissolution rate (Table 7 and Figure 5).

Powders (G, H, I, and J) floated immediately in the simulated gastric conditions, but tablets obtained through the direct compression of the same powders did not float for more than 15 min. This could be explained by increased density, as a result of compression during tablet manufacturing, which leads to slower surface wetting. Therefore, the influence of gas-generating agents (sodium bicarbonate, calcium carbonate) and hydrophilic polymers (hydroxypropyl methylcellulose, sodium alginate, and croscarmellose sodium) on the floating lag time was further investigated. The addition of sodium bicarbonate (7.5%) significantly reduced the floating lag time (Table 7). On the other hand, the floating lag time was longer than 15 min in the case of tablets prepared with the addition of calcium carbonate/hydroxypropyl methylcellulose/sodium alginate/croscarmellose sodium in the same concentration (7.5%). Also, it was noticed that tablets with higher content of sodium bicarbonate (10%) were not optimal since sticking between the punch and tablet was noted. Thapa and Jeong reported that with increasing amounts of sodium bicarbonate, the floating lag time decreased, but the mechanical (tensile) strength of tablets was also lower [37]. However, the friability of all tablet formulations with sodium bicarbonate (7.5%) was below 1%, indicating that tablets will be able to resist stresses during handling and transport (Table 7).

Therefore, formulations with 7.5% of sodium bicarbonate were selected as optimal and were further analyzed. It was noticed that trehalose content (*p* = 0.015) has a significant influence on floating lag time, and it was evident that with the increase in trehalose content, the floating lag time increases. This finding could be related to a high relative density of trehalose (1.22 g/cm^3^), which consequently affected the density of tablets. Also, homogenization time (*p* = 0.000) influenced the floating lag time significantly, and it was noticed that with increased HSH time, the floating lag time was longer, suggesting that the homogenization time affected lipid distribution and/or powder density and porosity (Figure 3G,H). Furthermore, it was noticed that all tablet formulations floated for more than 6 h.

Mucoadhesion enhances gastric retention by allowing the dosage form to adhere to the gastric mucosa, thereby resisting peristaltic clearance and providing extended contact with the absorption site. The developed floating gastroretentive tablets exhibited adhesion forces exceeding 1.05 N, while the influence of trehalose content (*p* = 0.609) and HSH time (*p* = 0.797) was not significant under biorelevant conditions (Table 7). A comparable formulation with allopurinol demonstrated in vivo gastric retention for 24 h when the tablet’s in vitro adhesive strength was 0.94 N, suggesting that the mucoadhesive strength observed in the investigated systems is sufficient for prolonged gastric residence. Nonetheless, it is important to note that mucus turnover, the presence of food, and variable gastric fluid volumes can influence mucoadhesion and floating ability. Dual-mechanism (floating plus mucoadhesion) formulations offer a more reliable approach to gastroretention because they mitigate the individual limitations of each mechanism. Consequently, these tablets are anticipated to maintain robust gastric retention across different physiological conditions (fasted versus fed states), ultimately enhancing the bioavailability of the encapsulated bioactive compounds [4,33].

Dissolution profiles of gentiopicroside from dry gentian extract and tablets made from SLM loaded with gentian extract and sodium bicarbonate are presented in Figure 5A. Gentiopicroside was dissolved from dry gentian extract powder rapidly (94.02% for 45 min). On the other hand, the investigated tablets are characterized by biphasic release, exhibiting the burst release of gentiopicroside in the first 15 min and sustained release in the second phase. The burst release in the first phase could be the consequence of the presence of gentiopicroside on the surface of particles and the porous structure of the SLM, while the sustained release in the second phase indicated that gentian extract was successfully encapsulated in the SLM. Moreover, it is possible that gentiopicroside migrated from the lipid matrix to the particle surface during compression, as a result of the mechanical forces applied during tableting. This hypothesis is supported by preliminary studies, which showed that powders of the same composition as the tablets do not exhibit burst release during in vitro release testing. Additionally, burst release in low-density gastroretentive systems has been previously linked to disruptions in system integrity [4], which may further explain the observed release behavior in the investigated tablets based on low-density solid lipid microparticles.

The same trend was evident in the case of tablets without sodium bicarbonate obtained using a similar technique. However, it is noticeable that sustained release was more pronounced in the case of formulations with sodium bicarbonate (G, H, I, and J) than in tablets without it [11]. It was reported that sodium bicarbonate could reduce the release rate of highly water-soluble active compounds, due to the formation of CO_2_, which is entrapped in the gel layer, and obstruct the diffusion of a bioactive compound from the matrix [37]. According to the presented dissolution profiles, tablets G and H, with a lower content of trehalose, were characterized by a slower release rate (Figure 5A). Moreover, trehalose content does not significantly affect the dissolution of gentiopicroside in the first 45 min (*p* = 0.925), while the positive influence of trehalose content (*p* = 0.000) on the gentiopicroside release rate was observed after 6 h (Figure 3I). It was previously reported that increasing the trehalose content positively affected the release of levonorgestrel from implantable PLGA microneedles [41]. Furthermore, it should be taken into account that the content of Gelucire^®^ 43/01, which is known as release-retarding agent, in tablet formulations G and H was higher than in tablet formulations I and J (Table 1). It is known that the release rate of bioactive compounds decreases with the increase in lipid content, due to increased thickness of the lipid matrix in SLM [73]. Presumably, the slower release rate was achieved in the case of tablet formulations G and H due to the lower trehalose and higher Gelucire^®^ 43/01 content.

Furthermore, according to the presented results, HSH time (*p* = 0.222) was not significant in the case of gentiopicroside release after 45 min. On the other hand, it was shown that with a longer homogenization process, the gentiopicroside release rate was lower after 6 h. It could be assumed that the homogenization process supported the incorporation of gentian extract in the matrix of particles and consequently significantly influenced (*p* = 0.050) the gentiopicroside release rate after 6 h (Figure 3J). It can be supposed that the longer duration of the W/O emulsion homogenization influenced the incorporation of the gentian extract into the W/O/W emulsions and, consequently, into the matrix of the particles, which enabled a slower dissolution of gentiopicroside.

In addition, according to the calculated similarity factors (Supplementary Table S1) it was confirmed that trehalose content and HSH time significantly influenced the gentiopicroside dissolution rate.

The Korsmeyer–Peppas model was the most appropriate model for describing the gentiopicroside release kinetics from all investigated tablet formulations (G, H, I, and J) according to the correlation coefficients (R^2^) calculated after the adjustment of dissolution profiles to different mathematical models (Supplementary Table S2). In addition, gentiopicroside was dissolved dominantly following the Fickian diffusion mechanism from all investigated tablet formulations. The release exponent (n) was below 0.45 for tablet formulation G, suggesting that gentiopicroside diffusion is proportional to the concentration (Fickian release). In the case of tablet formulations H, I, and J, the release exponent (n) was between 0.45 and 0.57, indicating the combined effect of diffusion and erosion mechanisms (anomalous transport). Generally, the Korsmeyer–Peppas model was used to describe Fickian diffusion from tablets based on non-swellable (lipid) matrices [74]. Tablets contained a high content of trehalose (20.20–33.66%), which is a water-soluble excipient, but the tablet did disintegrate during the in vitro release study because Gelucire^®^ 43/01 forms a non-swellable lipid matrix that limits water penetration and maintains tablet integrity. Additionally, sodium bicarbonate generates CO_2_, which can become entrapped in the lipid matrix, partially disrupting it and contributing to the anomalous (combined diffusion–erosion) release mechanism in formulations H, I, and J. Consequently, trehalose’s high solubility does not lead to full disintegration; rather, it facilitates a gradual release, largely controlled by diffusion through the lipid-based matrix. The observed anomalous transport in the case of tablets H–J can be related to the presence of sodium alginate and sodium bicarbonate, which could have affected the swelling and erosion of the system.

Furthermore, the size of formed nanoassociates during dissolution testing was in the range of 428.23 to 578.90 nm after 15 min, while after 6 h, it was slightly higher (Table 7). The homogenization time (*p* = 0.038) had a significant positive influence on the size of the formed nanoassociates after 15 min (Figure 3F), while the influence of trehalose concentration (*p* = 0.197) was not significant. The influence of homogenization time (*p* = 0.487) was not significant after 6 h, nor was the influence of trehalose concentration (*p* = 0.590). The PdI ranged from 0.517 to 0.620, which means that the distribution was wide and the drops had a non-uniform size. It was shown previously that compression during tableting could influence the uniformity of dispersed droplets [35]. The presence of the surfactant and lipid-based nanoassociates in the dissolution medium could be beneficial for a prospective increase in the absorption and, consequently, the bioavailability of the encapsulated bioactive compounds [75].

By incorporating a floating–mucoadhesive mechanism, we successfully developed gastroretentive tablets with a high yield and encapsulation efficiency that enables the modified release of the hydrophilic active compound. In contrast to other reported methods for mucoadhesive–floating delivery systems [6], the approach described in this study does not require organic solvent. Moreover, manufacturing procedures based on the incorporation of solid active compounds into mucoadhesive–floating systems have been employed previously [5,9], meaning that additional manufacturing steps to transform liquid plant extracts into a solid state are needed. On the other hand, the process described in this study directly incorporates the liquid extract via a double-emulsion/lyophilization process, enabling streamlining production and has a potential advantage for highly dosed hydrophilic compounds or liquid actives requiring gastric retention. This outcome compares favorably to existing technologies that often focus on smaller-dose or solid-state actives. Also, this study revealed that trehalose content and high-shear-homogenization time during W/O emulsion formation have a dominant impact on critical quality attributes, while the influence of other formulation and process parameters was not systematically evaluated here, which is a limitation. Future work could extend our QbD-based risk assessment to explore additional factors and strengthen the comparative data against alternative systems. Despite this, our methodology provides a robust, organic solvent-free platform for creating gastroretentive tablets, which may be adapted to other plant extracts or sensitive bioactive compounds.

Tablet formulation G was selected as optimal, since the viscosity of emulsion G was the lowest, as well as the moisture content in powder G. Furthermore, it was shown that powder G was characterized by a very high gentiopicroside encapsulation efficiency and high gentiopicroside content after 18 months. In addition, the content of gentian extract was higher in this formulation than in the case of formulations I and J.

Mucoadhesive force and floating lag time were in accordance with the defined requirements. Finally, the release of gentiopicroside was biphasic and controlled using a diffusion mechanism. Therefore, in order to assess the stability of optimal tablet formulation, the dissolution rate of tablet G was investigated after 18 months. The similarity factor f_2_ (72.10) indicated that the dissolution profile of tablet formulation G was not significantly different after 18 months of storage from the initial dissolution profile (Figure 5B).

The in vitro digestion of formulation G was investigated, since lipid-based formulations are substrates for digestive lipases and digestion can significantly influence their properties, i.e., the release of bioactive compounds. Gelucire^®^ 43/01 is extensively used as a lipid binder and release-retarding agent in gastroretntive formulations [10]. However, there are no reports about the digestion of lipid formulations with Gelucire^®^ 43/01. Furthermore, it is generally supposed that pancreatic lipase is the main enzyme involved in lipolysis, and consequently, the gastric step is omitted in most in vitro digestion models. However, it is known that 10–30% of the triglyceride lipolysis is catalyzed by gastric lipase [76], and therefore, this step could be important in the case of lipid-based gastroretentive formulations. The results of the digestion test for tablet formulation G are presented in Figure 5C. The influence of duodenal enzymes was evident, while the effect of gastric digestion was not significant. This result indicates that pancreatic lipase is the main enzyme involved in the digestion of tablet formulation G. In vitro digestion experiments demonstrated that optimal formulation resists gastric enzymatic attacks yet remains susceptible to intestinal lipases, indicating that any prematurely evacuated tablets would still release their load in the small intestine. It should be noted that the issue of premature evacuation was not addressed in previous studies.

Since it was observed that the tablets did not disintegrate during the gastric phase, the powder with the same composition as tablet formulation G was tested under the same conditions to investigate whether the physical barrier disabled the activity of gastric lipases. According to the results presented in Figure 5C, it was evident that the gastric phase significantly affected the digestion of the powder G. Thus, it is shown that formulated SLM powder with Gelucire^®^ 43/01, PGPR, and lecithin as lipid components was the substrate for the gastric lipases, but in the case of tablet G, this influence was not observed since the physical barrier disables the activity of gastric lipase.

## 4. Conclusions

In this study, gastroretentive lipid-based tablets loaded with gentian root extract obtained through the lyophilization of double emulsions were developed using a quality by design (QbD) approach employing elements such as quality target product profile (QTPP), critical quality attributes (CQAs), and risk assessment analysis (Ishikawa fish-bone diagram and risk estimation matrix). Furthermore, a robust, organic solvent-free method for obtaining dual-mechanism gastroretentive tablets could be adapted to other hydrophilic plant extracts or sensitive bioactive compounds that need to be retained in the stomach.

According to the risk assessment analysis, it was evident that the trehalose content and high-shear-homogenization time of W/O emulsion were factors with the highest impact on the investigated CQAs, and consequently, their influence was further examined. Trehalose content in the investigated range (3.94–7.88%) significantly affected the emulsions’ viscosity and, consequently, the drying process. It was shown that emulsions with lower trehalose content were characterized by lower viscosity, and thus, the moisture content of powders obtained after their lyophilization was lower. Trehalose content significantly influenced the encapsulation efficiency in a positive way. Furthermore, the floating lag time was shorter in the case of tablets obtained through the lyophilization of emulsions with lower trehalose content and during the shorter high-shear homogenization process. The homogenization time in the investigated range (3–6 min) had a positive effect on the encapsulation efficiency after 18 months, and it was observed that the dissolution rate of gentiopicroside from the tablets after 6 h was lower when the homogenization lasted longer. Also, the gentiopicroside release rate was significantly lower in the tablet formulations with lower trehalose and higher lipid content.

A powder formulation with optimal yield, high gentiopicroside encapsulation efficiency during 18 months, good flowability, and lowest moisture content and tablets with adequate floating lag time, mucoadhesive properties, and biphasic release were obtained through the lyophilization of double emulsion with 3.94% trehalose homogenized for 6 min during the processing of the O/W emulsion. In vitro digestion experiments demonstrated that the optimal formulation resists the influence of gastric enzymes while remaining susceptible to intestinal lipases, suggesting that any tablets prematurely evacuated from the stomach would still release their contents in the small intestine. Based on the in vitro results of the present study, it is anticipated that the optimal tablet formulation will ensure the prolonged release of the gentian root extract in the stomach, leading to an extended elimination half-life, improved bioavailability of gentiopicroside, and better patient outcomes. Future studies should focus on assessing the pharmacokinetic and pharmacodynamic profiles of the optimal tablet formulation in vivo.

## Figures and Tables

**Table 1:** Composition of emulsions (G, H, I, and J) and theoretical composition of powders (G, H, I, and J) after lyophilization.

		Emulsion			Powder	
		I and J	G and H		I and J	G and H
W1	Liquid gentian extract (%)	13.13	13.13	Dry gentian extract (%)	19.35	23.26
	Sodium chloride (%)	0.06	0.06	Sodium chloride (%)	0.25	0.30
	Sodium alginate (%)	0.26	0.26	Sodium alginate (%)	1.12	1.35
O	Gelucire^®^ 43/01 (%)	5.32	5.32	Gelucire^®^ 43/01 (%)	22.73	27.33
	PGPR (%)	0.98	0.98	PGPR (%)	4.21	5.06
W2	Purified water (%)	68.00	71.93	Purified water (%)	0	0
	Sodium chloride (%)	0.23	0.23	Sodium chloride (%)	0.99	1.19
	Sodium alginate (%)	1.58	1.58	Sodium alginate (%)	6.73	8.09
	Trehalose (%)	7.88	3.94	Trehalose (%)	33.66	20.20
	Lecithin (%)	1.58	1.58	Lecithin (%)	6.73	8.09
	Sylysia^®^ 350 (%)	1.00	1.00	Sylysia^®^ 350 (%)	4.23	5.09

W1—inner water phase; O—oil phase; W2—outer water phase; PGPR—polyglycerol polyricinoleate.

**Table 2:** Quality target product profile (QTPP) for lipid-based gastroretentive tablets.

QTPP	Target	Explanation
Route of administration	Oral	According to the EMA monograph [13], gentian root extract is administered via the oral route.
Indication	Dyspeptic symptoms/gastrointestinal disorders: heartburn, vomiting, stomach pain, nausea, loss of appetite, constipation, flatulence	Furthermore, antioxidant, anti-inflammatory, anti-microbial, anti-obesogenic, anti-atherosclerotic, gastroprotective, neurotrophic, anti-genotoxic effects are reported for gentian extracts, and isolated bioactive components [13,29].
Dosage form type	Gastroretentive tablets	The prolonged residence time of gentian extract in the stomach is desired due to the low bioavailability and short elimination half-life of gentiopicroside in conventional forms, as well as due to the local gastric effect of gentian extract [13,14].
Delivery type	Combined floating–mucoadhesive lipid-based system	The absorption of bioactive compounds is enhanced when triglyceride-based formulations are in contact with intestinal membranes. Also, the combination of the floating and mucoadhesive approach could improve gastroretention [2,12,30].
Dissolution profile	Modified gentiopicroside release	The release of an initial, effective dose of gentiopicroside, followed by a further sustained release at the place of action (stomach), is required in order to achieve and maintain the therapeutic effect of the gentian extract.
Stability	At least an 18-month shelf life	Store at room temperature.

**Table 3:** Critical quality attributes (CQA) and explanations.

	Target	Is This CQA?	Justification
Color Odor Appearance	Acceptable to patients	No	This parameter is not critical for efficacy and safety.
Emulsion viscosity	As low as possible minimal and maximal apparent viscosity	Yes	To encapsulate active compounds effectively in a dehydrated emulsion, an encapsulating matrix should have low viscosity [31].
Physical stability	No separation or change in consistency after the centrifugation test of double emulsion	No	It is essential that the double emulsions are physically stable. This parameter was not considered a critical parameter, since the tested formulations during the preliminary phase were stable.
Encapsulation efficiency	≥80%	Yes	As high as possible. A higher encapsulation efficiency is crucial since the manufacturing costs are reduced in this way. Furthermore, higher gentiopicroside content in the formulation can result in a reduced dosing frequency and consequently improved patient compliance.
Encapsulation efficiency after 18 months	≥90%	Yes	It is important to retain high content of gentiopicroside during storage.
Yield	≥80%	No	It is essential to develop powder with high yield in order to decrease production costs. This parameter is not considered critical since during preliminary studies, the yield of the investigated formulations was high.
Antioxidant potency composite index (ACI)—after 12 months	≥70%	No	It is vital to retain the high antioxidant activity of the extract during production and storage. However, this result could be affected by a number of factors, such as the type and amount of solvent used, the presence and concentration of hydrogen and metal ions, and the turbidity of the sample [32].
Moisture content	As low as possible	Yes	Moisture content is a critical attribute, since elevated water content could influence physico-chemical and microbiological stability, as well as flowability.
Flowability	Carr index ≤ 15 Hausner ratio ≤ 1.18	Yes	Free-flowing powders are more suitable for tablet production.
Floating duration (tablets)	≥6 h	No	As long as possible. This parameter is not considered critical since during preliminary studies, the investigated formulations floated for more than 6 h.
Floating lag time (tablets)	0–5 min	Yes	As minimum as possible, since increase in the floating lag time increases the chance of the gastric emptying of the tablet before the release of gentiopicroside in the stomach.
Gentiopicroside release	20% ≤ Q 45 min ≤ 40% 60% ≤Q 6 h ≤ 100%	Yes	It is reported that gentiopicroside elimination half-life is short (2.8 h) [14]. Therefore, it is important to achieve an initial, effective dose and, after that, to provide sustained release in order to increase bioavailability and to reduce the dosing frequency.
Friability of tablets	According to Ph. Eur. 11.0 (2023) requirements—less than 1%	No	This parameter is not considered critical since during preliminary studies, all investigated formulations accomplished the requirements.
Force of adhesion	More than 0.94 N	Yes	It is reported that mucoadhesive–floating tablets with a force of adhesion of 0.94 N enabled gastroretention in vivo [33].
Z average	≤1000 nm	Yes	The presence of smaller droplets during the in vitro release study may accelerate the release and improve the bioavailability of the encapsulated bioactive compounds [34,35].

**Table 4:** Risk estimation matrix (REM) for gastroretentive tablets presenting qualitative initial risk for material attributes and process parameters.

CQA	Material Atributes							Process Parameters								
	Lipid Content	W/O Emulsifier Content	O/W Emulsifier Content	Trehalose Content	Sylysia^®^ 350 Content	Sodium Alginate Content	Salt Content	Sirring Time	Stirring Speed	HSH Speed	HSH Time	Lyophilization Time	Lyophilization Pressure	Lyophilization Temperature	Compression Force	Dwell Time
Emulsion viscosity	Medium	Medium	Low	Medium	Low	High	Low	Low	Low	Medium	Medium					
Floatation lag time	Medium	Low	Low	Medium	Medium	Low	Low	Low	Low	Medium	Medium	Low	Medium	Low	High	Medium
Gentiopicroside release	High	Medium	Medium	High	Medium	Medium	Low	Low	Medium	Medium	High	Medium	Medium	Low	Medium	Medium
Force of adhesion	Low	Medium	Low	Low	Low	High	Low	Low	Low	Low	Low	Low	Low	Low	Low	Low
Encapsulation efficiency	High	High	High	High	Low	Low	Medium	Medium	Medium	High	High	Medium	Medium	Low	Low	Low
Encapsulation efficiency after 18 months	Medium	High	High	High	Low	Medium	Medium	Medium	Medium	High	High	Medium	Medium	Medium	Low	Low
Flowability	Medium	Low	Low	Medium	Medium	Low	Low	Low	Low	Low	Low	Medium	Low	Low	Low	Low
Moisture content	Low	Low	Low	High	Medium	Low	Low	Low	Low	Low	Medium	High	Medium	Medium	Low	Low
Z-ave	Low	Medium	Medium	Medium	Low	Low	Low	Low	Medium	Medium	Medium	Medium	Medium	Low	Medium	Medium

CQA—critical quality attributes; HSH—high shear homogenization.

**Table 5:** Conductivity, pH, maximal apparent viscosity (η_max_, at 6.9 s^−1^), minimal apparent viscosity (η_min_, at 200 s^−1^), and hysteresis area (HA) for the double emulsions of prepared double emulsions (G–J).

	pH	Conductivity (µS/cm)	η_max_ (Pa·s)	η_min_ (Pa·s)	HA (Pa/s)
G	4.46 ± 0.18	4.25 ± 0.32	4.17 ± 0.02	0.424 ± 0.007	2963.45
H	4.39 ± 0.11	5.54 ± 0.28	6.21 ± 0.07	0.478 ± 0.008	3997.54
I	4.39 ± 0.09	5.21 ± 0.24	7.37 ± 0.30	0.600 ± 0.044	4932.91
J	4.40 ± 0.13	4.78 ± 0.17	6.60 ± 0.06	0.610 ± 0.006	4815.73

**Table 6:** Gentiopicroside encapsulation efficiency, Carr index, Hausner ratios, moisture content, floating lag time, floating duration, and antioxidant potency composite index of prepared powders (G, H, I, and J).

	Encapsulation Efficiency (%)	Encapsulation Efficiency- 18 Months (%)	Yield (%)	Carr Index (%)	Hausner Ratio	Moisture Content (%)	Floating Lag Time (s)	Floating Duration (h)	ACI- 12 Months (%)
G	95.13 ± 0.68	94.34 ± 1.31	92.31 ± 2.11	13.53 ± 0.23	1.16 ± 0.01	6.23 ± 1.04	0	>6	89.82 ± 2.85
H	80.35 ± 1.36	90.33 ± 1.22	94.88 ± 3.16	12.92 ± 0.42	1.14 ± 0.02	7.16 ± 0.52	0	>6	95.20 ± 3.94
I	94.72 ± 0.75	93.64 ± 1.23	90.56 ± 1.79	13.50 ± 0.51	1.16 ± 0.01	7.70 ± 0.41	0	>6	89.61 ± 3.01
J	97.34 ± 0.29	92.24 ± 1.40	92.64 ± 1.94	13.35 ± 0.34	1.15 ± 0.02	8.00 ± 0.63	0	>6	86.45 ± 4.23

**Table 7:** Friability, floating lag time, floating duration, and mucoadhesive force of investigated tablets (G, H, I, and J), as well as size (Z-average) of dispersed nanostructures measured after 15 min and after 6 h during in vitro release testing.

	Friability (%)	Floating Lag Time (s)	Floating Duration (h)	Force of Adhesion (N)	Z-average (nm)— 15 min	Z-average (nm)— 6 h
G	0.51	275 ± 12	>6	1.18 ± 0.35	521.40 ± 71.99	529.70 ± 6.30
H	0.43	220 ± 10	>6	1.23 ± 0.41	428.23 ± 45.68	642.47 ± 16.93
I	0.60	284 ± 12	>6	1.05 ± 0.33	578.90 ± 112.81	707.60 ± 24.94
J	0.61	251 ± 7	>6	1.12 ± 0.54	480.45 ± 29.77	521.27 ± 18.53

## Data Availability

The data presented in this study are available on request from the corresponding author.

## Supplementary Materials

The following Supporting Information can be downloaded at https://www.mdpi.com/article/10.3390/pharmaceutics17010071/s1: Table S1. Similarity factor for investigated tablet formulations (G, H, I, and J). Table S2. Gentiopicroside release kinetics (coefficient of determination R^2^ and release exponent n) from tablets. Figure S1. Photomicrographs of (A) double emulsion G; (B) double emulsion H; (C) double emulsion I; (D) double emulsion J. Figure S2. (A) FTIR spectra of gentian extract, SLM powders (G, H, I and J), and Gelucire^®^ 43/01. (B) DSC thermograms for gentian extract and SLM (G, H, I and J) powders.
