# Supplementary material for: Dual-Mechanism Gastroretentive Tablets with Encapsulated Gentian Root Extract
Source: Pharmaceutics. 2025 Jan 7;17(1):71. doi: 10.3390/pharmaceutics17010071 (PMC11768846; doi:10.3390/pharmaceutics17010071)

Supplementary

Table S1. Similarity factor for investigated tablet formulations (G, H, I, and J).

	f_2 (similarity factor)
G vs H	54.40
G vs I	38.35
G vs J	36.13
H vs I	50.87
H vs J	47.82
I vs J	68.00

Table S2. Gentiopicroside release kinetics (coefficient of determination R2 and release exponent n) from tablets.

	Correlation Coefficients (r^2)				n
	Zero-order	First-order	Higuchi	Krosmayer-Peppas	
G	0.8778	0.8558	0.9925	0.9960	0.4061
H	0.8909	0.8190	0.9971	0.9976	0.4607
I	0.9075	0.8070	0.9828	0.9881	0.5787
J	0.8826	0.7862	0.9901	0.9916	0.5217

Figure S1. Photomicrographs of (A) double emulsion G; (B) double emulsion H; (C) double emulsion I; (D) double emulsion J.

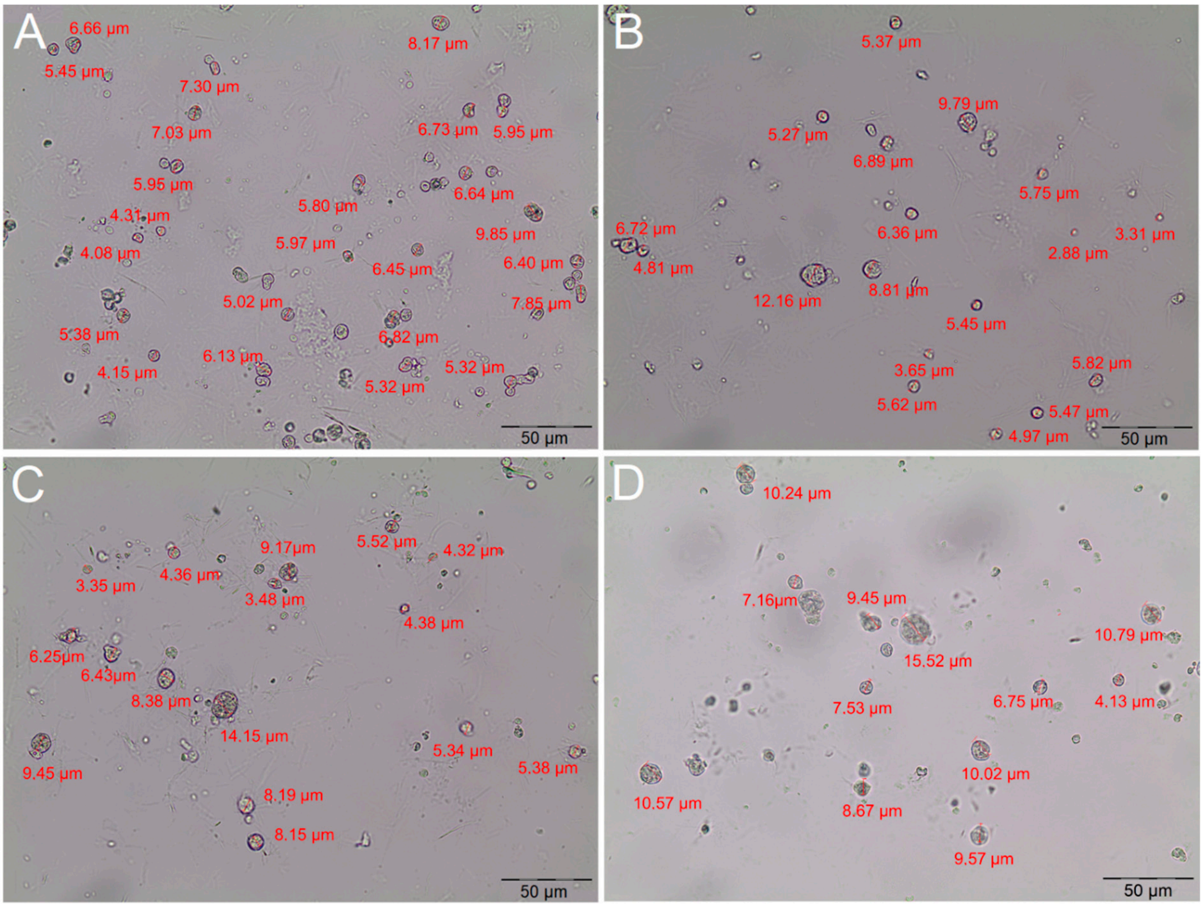

Figure S2. (A) FTIR Spectra of gentian extract, SLM powders (G, H, I and J), and Gelucire® 43/01; (B) DSC thermograms for gentian extract and SLM (G, H, I and J) powders.

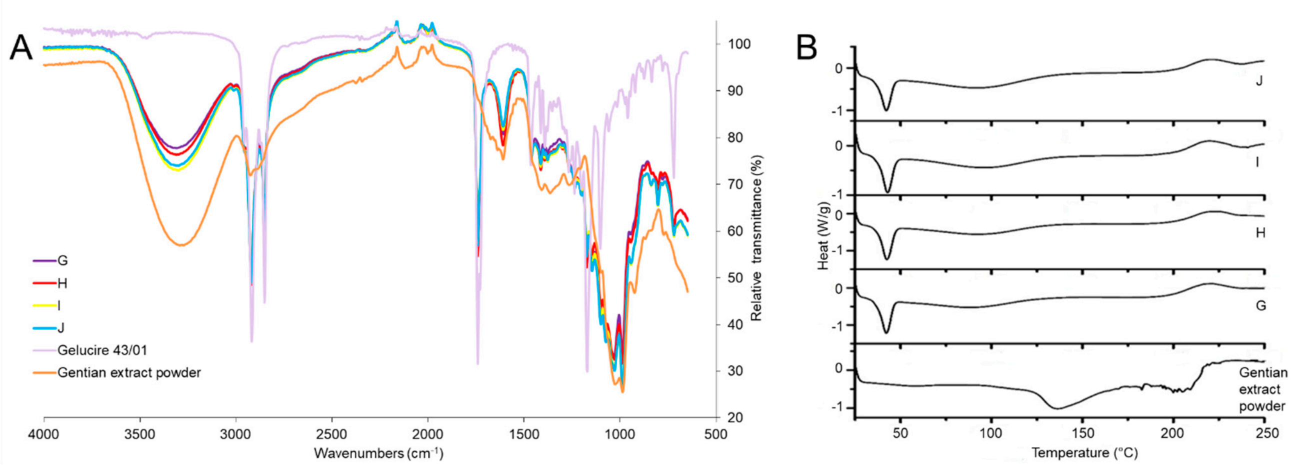

Supplement: Supplementary file 1 [file pharmaceutics-17-00071-s001.zip › pharmaceutics-3374520-supplementary.pdf]
